# Diffuse pulmonary calcifications: A case series and review of literature

**DOI:** 10.1002/rcr2.839

**Published:** 2021-08-31

**Authors:** Amir Jarjou'i, Naama Bogot, George Kalak, Chen Chen‐Shuali, Ariel Rokach, Gabriel Izbicki, Nissim Arish

**Affiliations:** ^1^ Pulmonary Institute, Department of Medicine Shaare Zedek Medical Center Jerusalem Israel; ^2^ Faculty of Medicine Hebrew University of Jerusalem Jerusalem Israel; ^3^ Radiology Department Shaare Zedek Medical Center Jerusalem Israel

**Keywords:** dystrophic pulmonary calcifications, metastatic pulmonary calcifications, pulmonary alveolar microlithiasis, pulmonary calcifications

## Abstract

Pulmonary calcifications are usually incidental asymptomatic findings discovered on x‐rays or computed tomography scans that can be easily overlooked, and their significance undermined, especially in a seemingly asymptomatic person. Calcifications can be a marker of chronicity or disease severity, and thus have diagnostic value. Rarely, calcification can be the direct cause of morbidity. Calcifications can be either localized or diffuse. Many diseases, in particular infectious diseases, can cause localized calcifications. Diffuse calcifications are less common and usually secondary to a handful of conditions such as dystrophic pulmonary calcifications, metastatic pulmonary calcifications, disseminated pulmonary ossifications and pulmonary alveolar microlithiasis. We describe three cases of diffuse pulmonary calcifications, review the different causes of diffuse pulmonary calcifications and provide some indicators on how to differentiate between them. Differentiating between the different types of pulmonary calcifications has significant implications on the management and prognosis of the patients, and thus it is important to distinguish between them.

## Graphical abstract

Pulmonary calcifications are usually incidental asymptomatic findings discovered on x‐rays or computed tomography scans that can be easily overlooked, and their significance undermined, especially in a seemingly asymptomatic person. We describe three cases of diffuse pulmonary calcifications, review the different types of diffuse pulmonary calcifications and provide clinical as well as radiological clues to establishing a diagnosis.

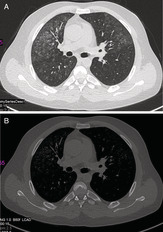

## INTRODUCTION

Pulmonary calcifications can be either localized or diffuse. Localized calcifications are much more common and are usually of diagnostic value only. Diffuse calcifications on the other hand can be the direct cause of morbidity; however, they are often overlooked. There are many causes of pulmonary calcifications, the most common being infectious diseases. Other causes include inflammatory, fibrotic, genetic, metabolic, neoplastic and cardiac diseases.^1^, ^2^, ^3^, ^4^, ^5^ It can be challenging and time‐consuming to differentiate between the different aetiologies. We describe three cases of diffuse pulmonary calcifications, review the literature on the different types and provide clinical as well as radiological clues to establishing a diagnosis.

## CASE SERIES

### Case 1

A 40‐year‐old male patient presented to the emergency room with shortness of breath, dry cough and low‐grade fever for 1 month. He is a heavy smoker with about 40 pack‐years.

He had kidney transplantation 10 years ago, following renal failure secondary to focal segmental glomerulonephritis. He is treated with prednisone, tacrolimus and mycophenolic acid.

He underwent a chest x‐ray that showed bilateral opacities.

**FIGURE 1:**
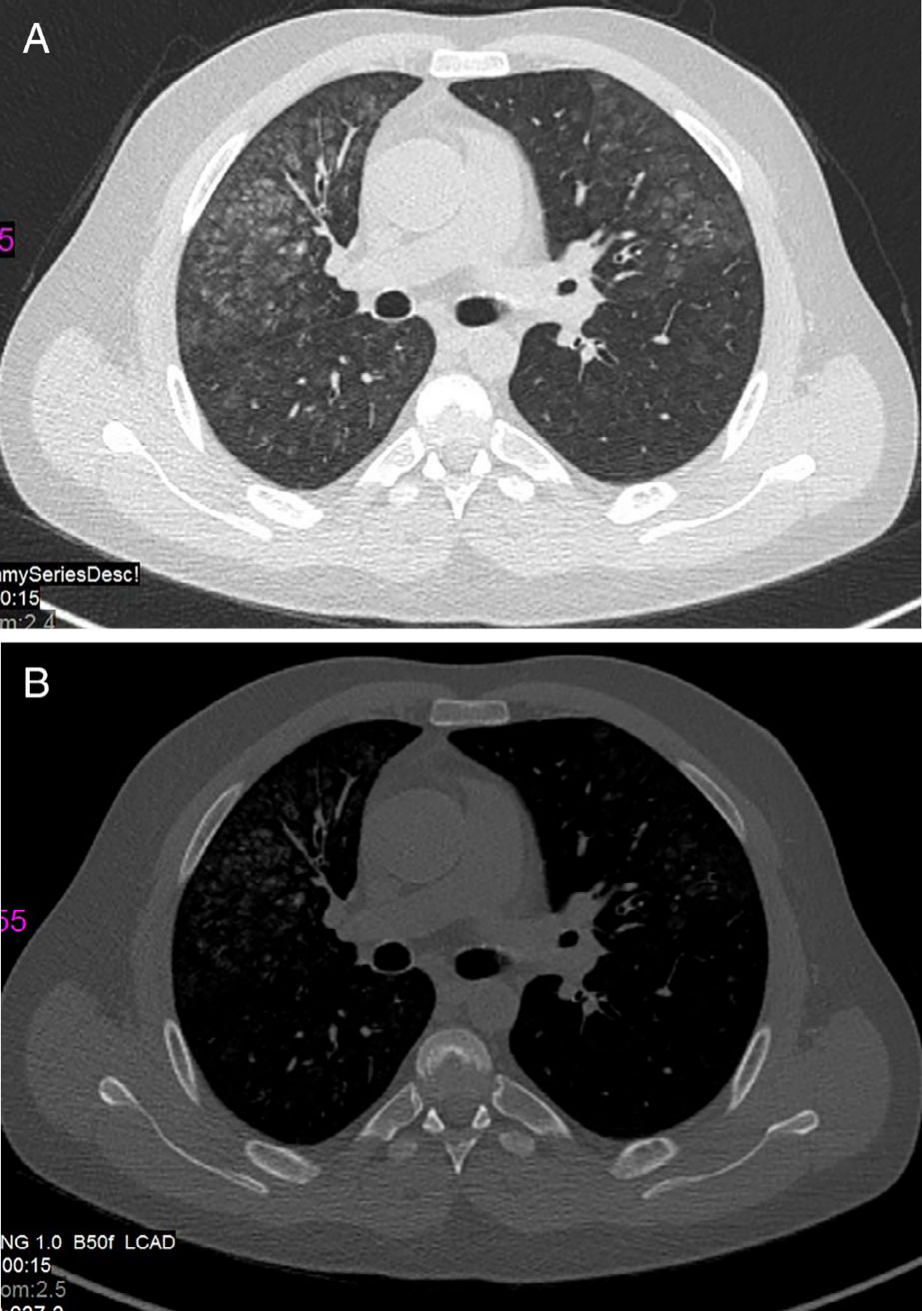
Case 1: A 40‐year‐old male with chronic renal failureand diagnosis of metastatic pulmonary calcification. (A) High‐resolution computed tomography shows upper lungs predominant fluffy‐appearing centrilobular ground‐glass opacities. (B) Bone windows show no apparent calcifications

A chest computed tomography (CT) scan showed bilateral ground‐glass opacities, mainly peripheral and more prominent in the upper lobes with no apparent calcifications. The radiological pattern and distribution were suggestive of metastatic pulmonary calcifications (MPC) (shown in Figure 1).

Due to his immunocompromised state and his low‐grade fever, the patient underwent bronchoscopy with bronchoalveolar lavage (BAL) and transbronchial biopsies that ruled out infection and confirmed the initial diagnosis of MPC. His dialysis protocol was changed with better control of his calcium and phosphate levels. He has had yearly follow‐ups for 3 years since then. A recent chest x‐ray showed marked regression of the ground‐glass opacities, and his recent pulmonary function test (PFT) was normal. He still complains of mild, occasional dry cough and still smokes despite medical advice.

### Case 2

An 83‐year‐old asthmatic male was suffering from exertional dyspnoea in the 3 months before his referral.

On auscultation, the patient had wheezes and prolonged expiration bilaterally.

**FIGURE 2:**
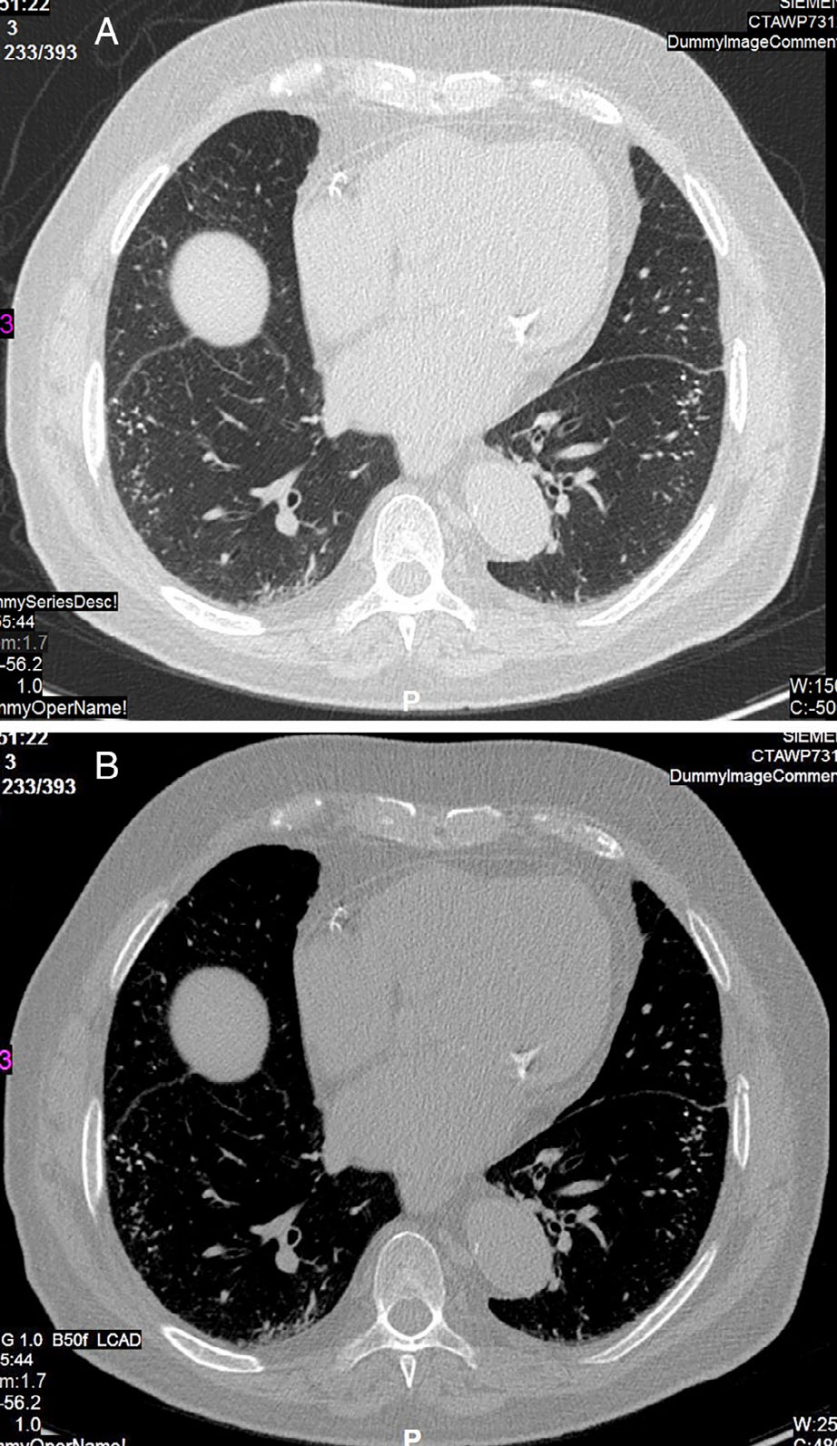
Case 2: An 83‐year‐old male with disseminated pulmonary ossification and fibrotic lung changes. (A) High‐resolution computed tomography using lung window settings demonstrated branching dendritic calcifications mainly in the lower lobes with mild reticular changes. (B) In bone windows, fine calcifications are seen

Chest CT scan showed branching, dendritic, calcifications mainly in the lower lobes with mild fibrotic changes. The clinical and radiological presentation was suggestive of disseminated pulmonary ossification (DPO) of the dendritic form (shown in Figure 2).

The patient's dyspnoea improved after optimizing his asthma treatment. Since then, the patient has been followed up regularly with no apparent deterioration in his condition. His PFT showed a mild obstructive pattern with reversibility.

### Case 3

A 93‐year‐old female was admitted to the internal medicine ward due to shortness of breath, dry cough and hypoxia with a 7‐day duration. The patient had a history of ischaemic heart disease, severely impaired left ventricle contraction and diastolic dysfunction grade II. She had a long‐standing diagnosis of silicosis in her medical records based on an ambulatory CT scan done many years ago.

**FIGURE 3:**
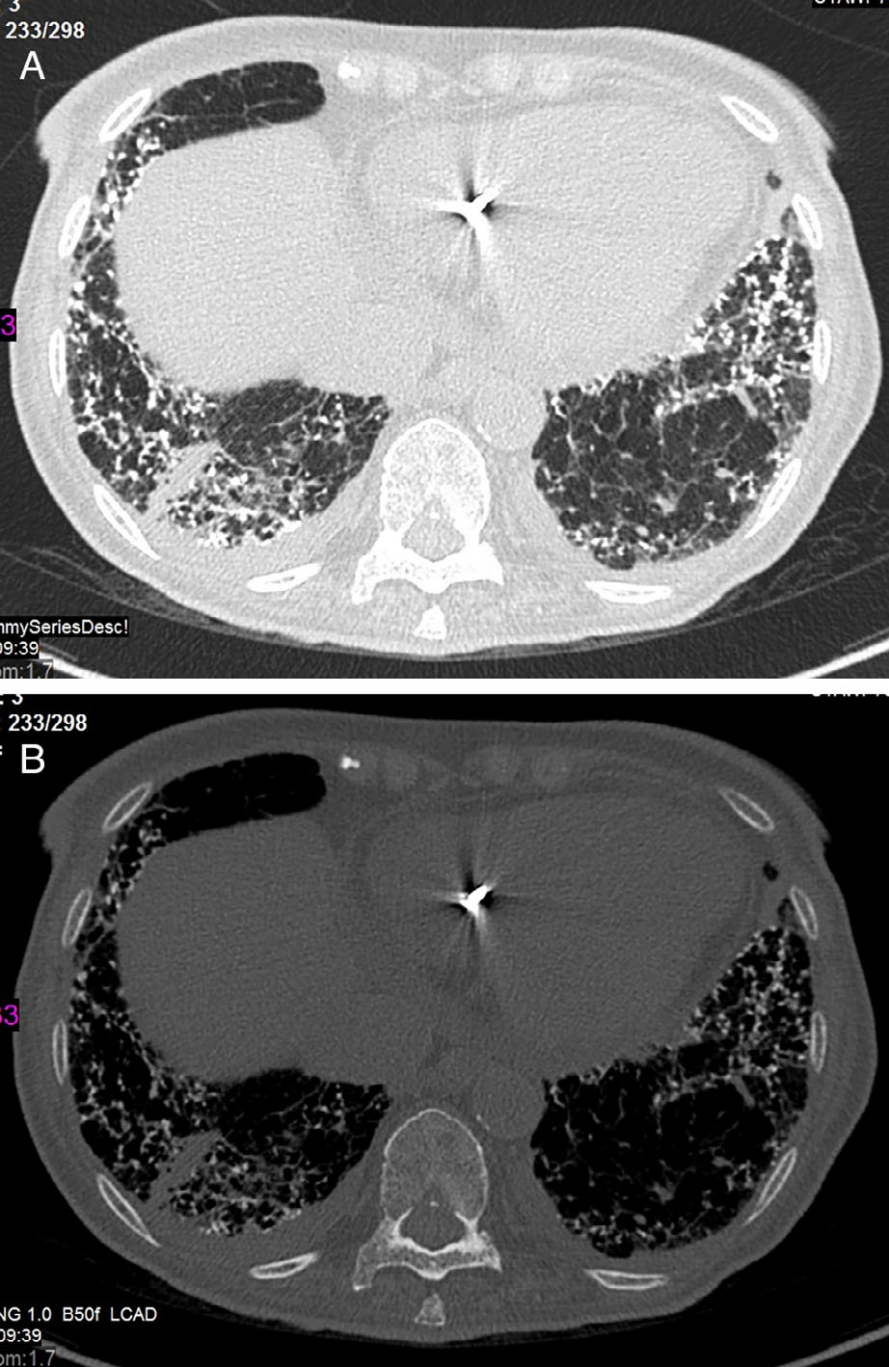
Case 3: A 93‐year‐old female with disseminated pulmonary ossification and chronic heart failure. (A) High‐resolution computed tomography shows nodular calcifications along the septa predominantly in the lower lobes. (B) On bone window setting, the calcifications are more conspicuous

A CT scan was done upon her admission and showed diffuse, lower lobe predominant, nodular calcifications with signs of pulmonary congestion. There was no mediastinal lymph node calcification or enlargement. The radiological findings were consistent with nodular DPO and pulmonary congestion rather than silicosis (shown in Figure 3). The patient ruled out any exposure to silica or other dust. In comparison to the previous CT scan, an evident progression was seen. Her PFT showed a restrictive pattern, with total lung capacity of 58% of the predicted value and a diffusion capacity of carbon monoxide of 37% of the predicted value.

The patient's acute symptoms subsided after appropriate diuretic treatment. She was referred to a cardiologist for further optimization of her heart failure treatment.

## DISCUSSION

Pulmonary calcifications involve the lung parenchyma. They can be categorized depending on the mechanism, form, site and extent (localized vs. diffuse). We focused on diffuse pulmonary calcification. Localized pulmonary calcification as well as pleural and lymph node calcifications are not reviewed in this paper.

Pulmonary calcifications are considered diffuse when there are multiple foci and involvement of both lungs or multiple lobes. There are four main mechanisms of diffuse calcifications—dystrophic pulmonary calcifications, MPC, DPO and pulmonary alveolar microlithiasis (PAM).^1^, ^2^, ^3^, ^4^, ^5^

### Dystrophic pulmonary calcifications

Dystrophic calcifications are the most common cause of diffuse pulmonary calcifications. Infectious and occupational aetiologies are the leading causes.^1^, ^2^, ^3^, ^4^, ^5^

Radiological features of dystrophic calcifications include signs of previous tissue damage (e.g., post‐infectious granulomas or metastasis), lymph node enlargement or calcification and pleural calcification, thickening or plaques. Other features that help us distinguish dystrophic calcifications from the other three aetiologies are the size, amount and distribution of calcifications. In dystrophic calcifications, multiple large calcified nodules are usually seen in comparison to the smaller, much more diffuse calcifications seen in MPC, DPO and PAM.^1^, ^2^, ^3^, ^4^, ^5^

Diagnosis is based on previous medical history and radiological features. A medical history of infectious diseases or exposure to dust is usually evident.

Prognosis depends on the underlying disease; calcifications in DPO are of diagnostic value but do not usually cause morbidity or mortality. Progression of the calcifications is not usual unless there is ongoing tissue damage and repair.

Treatment: In infectious diseases, no treatment is usually warranted unless signs of active disease are evident. In other aetiologies, the treatment is directed towards the underlying disease (e.g., stopping occupational exposure to the causative dust).

### Metastatic pulmonary calcifications

MPC is a metabolic lung disease characterized by the deposition of calcium in the pulmonary parenchyma.^1^

MPC occurs most often in association with conditions that directly or indirectly result in hypercalcaemia, for example, hyperparathyroidism and chronic renal failure. It rarely occurs in patients with normal renal function, normal calcium and phosphate levels and no underlying pulmonary disease. Either benign or malignant diseases can cause MPC.^1^, ^2^, ^3^, ^4^, ^5^, ^6^, ^7^ Orthotropic liver transplantation is a well‐documented cause of MPC.

Chronic renal failure and chronic dialysis are the most common causes of MPC. MPC is the most likely cause of multifocal pulmonary parenchymal calcification in patients with chronic renal failure.^7^ Due to improved dialysis techniques and control of calcium and phosphate levels, the incidence of MPC is declining.^8^

In MPC, calcifications usually occur in tissues that experienced significant changes in pH, such as the lungs, kidneys and stomach. In the lungs, the areas with the more alkaline media are more severely affected. The upper lobes and the peripheral zones have higher ventilation‐to‐perfusion ratio and thus have a more alkaline pH and are more commonly affected with MPC.^8^

Calcium deposition can be seen in the alveoli, alveolar septa, alveolar‐capillary walls, bronchial walls and small pulmonary vessels.

The most common radiological findings are centrilobular ground‐glass opacities, with numerous fluffy and poorly defined nodules measuring 3–10 mm with peripheral calcifications. Other patterns can be seen. In 40% of the cases, calcifications can be so minute that they do not show on the CT scans (Table 1).^1^, ^2^, ^3^, ^4^, ^7^

**TABLE 1:** HRCT findings in diffuse pulmonary calcifications

	Usual distribution	Usual findings	Associated findings	Unusual findings—may suggest alternate diagnosis	Causes
Dystrophic	Upper	Multiple calcified nodules usually >5 mm size	Tree in bud Bronchial wall thickening Lung masses Lymph node calcifications/enlargement Pleural thickening, plaques or calcifications Spleen or liver calcifications	Micronodules GGOs Dense calcifications	I. Infectious: ‐Tuberculosis ‐Mycobacteria ‐Varicella ‐Histoplasmosis ‐Pneumocystis carinii ‐Other infections II. Tumours: ‐Hamartomas ‐Sarcomas ‐Giant cell tumours of bone ‐Adenocarcinoma ‐Thyroid malignancy ‐Choriocarcinomas III. Occupational: ‐Silicosis ‐Coal workers pneumoconiosis ‐Siderosis ‐Stannosis ‐Baritosis IV. Miscellaneous: ‐Amyloidosis ‐Alveolar haemorrhage ‐Haemosiderosis ‐Sarcoidosis ‐Pulmonary infarction
MPC	Upper Peripheral	Calcifications only in 60% of cases! Three main patterns: 1. Centrilobular GGO, with numerous fluffy and poorly defined nodules measuring 3–10 mm + peripheral calcifications (most common) 2. Multiple calcified nodules 3. High‐attenuation consolidation with lobar distribution + calcification	Calcification of: Chest wall vessels Small pulmonary arteries Myocardium Bronchial walls Superior vena cava	Tree in bud Bronchial wall thickening Interlobular wall thickening Lymph node calcifications /enlargement Spleen or liver calcifications	Benign: I. Common aetiologies: ‐Chronic renal failure ‐Secondary hyperparathyroidism ‐Orthoptic (allogeneic) liver transplantation ‐Kidney transplantation II. Rare aetiologies: ‐Primary hyperparathyroidism ‐Excess exogenous administration of calcium and vitamin D ‐Sarcoidosis ‐Milk‐alkali syndrome ‐Osteoporosis ‐Osteitis deformans Malignant: I. Common aetiologies: ‐Massive osteolysis from metastases ‐Multiple myeloma ‐Parathyroid carcinoma II. Rare aetiologies: ‐Leukaemia ‐Lymphoma ‐Breast carcinoma ‐Synovial carcinoma ‐Choriocarcinoma ‐Malignant melanoma ‐Hypopharyngeal squamous carcinoma
DPO—nodular form	Lower	Densely calcified, smooth, round nodules of 1–5 mm size	Pulmonary congestion	Vascular involvement Bronchial wall thickening Tree in bud Lymph node calcifications /enlargement Spleen or liver calcifications	Mitral valve stenosis Chronic pulmonary oedema Pulmonary venous hypertension
DPO—dendriform	Lower	Branching lines of calcifications of 1–4 mm width in a bronchovascular distribution	Pulmonary fibrosis of varying degrees		Interstitial fibrosis Chronic inflammation
PAM	Lower Medial Posterior	Diffuse micronodules of 0.01–1 mm Four stages First: Precalcific, HRCT is non‐diagnostic. Calcifications are not readily seen A Tc‐99m scan can help in early detection Second: Classic sandpaper/sandstorm appearance Usually in ages 10–20 Third: More granular pattern with more diffuse opacifications Crazy‐paving pattern Fourth: White lungs—with dense diffuse calcifications	Calcifications along the interlobular septa with thickening Black pleura sign Calcification in other organs: Mammary glands Small intestine Kidneys Pancreas Ovaries/testes Placenta Prostate	Vascular involvement Bronchial wall thickening Tree in bud Lymph node calcifications /enlargement Spleen or liver calcifications	Mutations of the *SLC34A2* gene

Abbreviations: DPO, disseminated pulmonary ossification; GGO, groundWe have treated the expansion for "GGO" as "ground‐glass opacity". Please check and confirm if this is appropriate.‐glass opacity; HRCT, high‐resolution computed tomography; MPC, metastatic pulmonary calcification; PAM, pulmonary alveolar microlithiasis.

Diagnosis is based on typical radiological features in predisposed patients. Bronchoscopy with BAL and lung biopsies can sometimes be needed to confirm the diagnosis, as seen in Case 1.

Calcifications in MPC are progressive and can cause marked morbidity and symptoms; proper treatment can lead to significant regression of the opacities and the symptoms.

Treatment is usually directed towards better control of calcium and phosphate levels. In the case of haemodialysis, this usually includes changing the patient's medications and dialysis plan. In other aetiologies, the treatment is directed to the underlying cause.^6^, ^7^, ^8^

### Disseminated pulmonary ossifications

DPO is a chronic progressive, metaplastic ossification that is characterized by new bone formation with or without marrow elements. It can be idiopathic or secondary (more common). It can be secondary to mitral valve disease, chronic pulmonary congestion, pulmonary fibrosis disorders and amyloidosis. New bone tissue formation can occur in the alveolar spaces and thus will have a circular nodular form, or it can occur in the alveolar interstitium and septa, resulting in a branching dendriform appearance (Table 1).^1^, ^4^, ^7^, ^9^, ^10^, ^11^, ^12^

Nodular DPO is the more common type. It usually affects the alveolar spaces, preferentially the lower lobes. It is more common in passive congestion, such as in mitral valve stenosis, chronic pulmonary oedema and pulmonary venous hypertension.^1^, ^9^, ^10^, ^11^, ^12^

Dendriform DPO commonly affects the alveolar interstitium and the septa. It usually occurs in the setting of chronic inflammation and interstitial fibrosis. It typically appears as fine, dendritic, branching calcifications prominently in the lower lobes.^1^, ^9^, ^10^, ^11^, ^12^

Diagnosis is based on typical radiological features in a patient with a predisposing condition.

Calcifications can be progressive; however, they are usually a marker of the underlying disease's chronicity or progression. Morbidity and mortality are dependent on the underlying cause.

Treatment is based on managing the underlying disease.

### Pulmonary alveolar microlithiasis

PAM is a rare autosomal recessive genetic disorder characterized by calcifications within the alveoli. Mutations of the *SLC34A2* gene, which encodes a type IIb sodium‐phosphate cotransporter, result in intra‐alveolar accumulation of phosphate, which causes calcium deposition, leading to microliths.^13‐15^

The typical radiological feature is a ‘sandstorm’ appearance on chest x‐rays, characterized by diffuse micronodules of 0.01–0.1 mm in size, with calcifications usually starting and are more severe in the lower lobes, mainly in the medial and posterior parts (Table 1).^13^, ^14^

The typical radiological features in a person with a family history of PAM are diagnostic. In this case, a bronchoscopy is done; the common findings are microliths, which are periodic acid‐Schiff positive in the BAL and variable degrees of fibrosis with alveolar spaces filled with microliths in the lung biopsy.

PAM is a progressive disease; the rate of deterioration is variable among patients. Smokers tend to have more severe clinical phenotypes. PFT, as well as clinical assessment, help follow‐up patients with PAM, and detect early deterioration in lung function.^13^, ^14^, ^15^

Lung transplantation is the only treatment for advanced PAM. Clinical and radiological follow‐up as well as PFTs are essential in following up patients with PAM.^15^ Family members should be screened with chest x‐rays.^13^, ^14^

### Differentiating between types of diffuse pulmonary calcifications

Proper medical history, including occupational exposure, along with physical examination, is the cornerstone of establishing a diagnosis.

The extent, distribution, form and size of the calcifications are the main radiological features to consider (Table 1). Associated features are also important clues to the diagnosis, and they include:

- Calcifications of lymph nodes, pleura, blood vessels and calcifications of the myocardium, liver, spleen, stomach and kidneys.
- Signs of an underlying disease, such as fibrosis, granulomas, masses and pulmonary congestion.

It is essential to distinguish calcification from high‐attenuation opacities, for these are two different processes and are usually caused by different diseases.

## CONCLUSION

We conclude it is essential to distinguish between the different forms of, and entities causing, diffuse pulmonary calcifications. This differentiation has a significant impact on workup and treatment, including the need for further investigation, change in treatment plans and rarity in screening for family members (Table 2).

**TABLE 2:** Clinical implications of diffuse pulmonary calcifications

	Prognosis	Treatment	Screening	Diagnostic value
Dystrophic	Depends on the underlying cause	Directed to primary disease	No	Differentiating between the causes and treating accordingly
MPC	Poor in severe cases	Calcium/phosphate level control	No	Must be differentiated from opportunistic infections Prevent lung fibrosis
DPO	Depends on the cause	Depending on the cause	No	Can contribute to morbidity and symptoms Prevent/decrease ongoing lung damage
PAM	Poor	Lung transplant	Chest x‐ray for family members	Screening family members Timing for a lung transplant

Abbreviations: DPO, disseminated pulmonary ossification; MPC, metastatic pulmonary calcification; PAM, pulmonary alveolar microlithiasis.

## CONFLICT OF INTEREST

None declared.

## AUTHOR CONTRIBUTION

All authors have contributed substantially to the study design, data acquisition and drafting of the manuscript, and have read and approved this final version.

## ETHICS STATEMENT

Appropriate written informed consent was obtained for publication of this case series and accompanying images.
